# Patterns of Interspecific Variation in the Heart Rates of Embryonic Reptiles

**DOI:** 10.1371/journal.pone.0029027

**Published:** 2011-12-13

**Authors:** Wei-Guo Du, Hua Ye, Bo Zhao, Ligia Pizzatto, Xiang Ji, Richard Shine

**Affiliations:** 1 Key Laboratory of Animal Ecology and Conservational Biology, Institute of Zoology, Chinese Academy of Sciences, Beijing, China; 2 School of Biological Sciences A08, University of Sydney, New South Wales, Australia; 3 College of Biological and Environmental Sciences, Hangzhou Normal University, Hangzhou, China; 4 College of Life Sciences, Nanjing Normal University, Nanjing, Jiangsu, China; University of Sao Paulo, Brazil

## Abstract

New non-invasive technologies allow direct measurement of heart rates (and thus, developmental rates) of embryos. We applied these methods to a diverse array of oviparous reptiles (24 species of lizards, 18 snakes, 11 turtles, 1 crocodilian), to identify general influences on cardiac rates during embryogenesis. Heart rates increased with ambient temperature in all lineages, but (at the same temperature) were faster in lizards and turtles than in snakes and crocodilians. We analysed these data within a phylogenetic framework. Embryonic heart rates were faster in species with smaller adult sizes, smaller egg sizes, and shorter incubation periods. Phylogenetic changes in heart rates were negatively correlated with concurrent changes in adult body mass and residual incubation period among the lizards, snakes (especially within pythons) and crocodilians. The total number of embryonic heart beats between oviposition and hatching was lower in squamates than in turtles or the crocodilian. Within squamates, embryonic iguanians and gekkonids required more heartbeats to complete development than did embryos of the other squamate families that we tested. These differences plausibly reflect phylogenetic divergence in the proportion of embryogenesis completed before *versus* after laying.

## Introduction

The central biological significance of metabolic rate has spawned a rich literature on this topic, in a wide range of animals from insects to mammals [1], [2], [3]. Broad comparisons across the animal kingdom have stimulated general models to explain sources of variation in metabolic rate [4]. However, these studies mainly focus on post-embryonic stages of the life-history; much less attention has been paid to embryos [5], [6]. That focus partly reflects logistical issues (it is easier to measure mass-specific metabolic rates of adults than of embryos), but also the traditional view that embryos are simply organisms-in-progress, passively responding to ambient conditions. Hence, there has been less interest in interpreting the adaptive significance of traits manifested by embryos than in interpreting the corresponding traits in free-living stages of the life-history. Challenging that view, recent studies have argued that embryos are far from passive; instead, they show a range of adaptive responses to environmental challenges (e.g. behavioural thermoregulation, thermal acclimation: [7], [8], [9]). Metabolic rates of embryos thus are of great interest, with the potential to clarify the fundamental issue of how embryos adapt to their surroundings.

In oviparous amniotes, it is methodologically difficult to measure the physiological functioning of embryos that are enclosed within eggshells. The most widely used measure of embryonic metabolic state is the rate of oxygen consumption, which correlates in interesting ways with variables such as egg mass and incubation temperature [5]. Recent methodological advances in non-invasive monitoring provide an opportunity to measure another indicator of embryonic metabolic rate: heart rate [10], [11], [12]. Unlike oxygen consumption rates, heart rates are relatively independent of embryonic mass (and hence, constant through most of the incubation period [10], [11], [13], [14]); and thus, the eggs do not have to be destroyed to measure the embryonic masses associated with specific readings of heart rate. Heart rate is an important component of cardiac function, and highly correlated with oxygen consumption rate at least within species [15], [16], [17], [18]. Our newfound ability to non-invasively measure embryonic heart rates provides an exciting opportunity to explore the physiological adaptations of embryos [18].

Heart rate can be influenced by both intrinsic and extrinsic factors. For example, heart rates of embryos are significantly affected by mode of development (altricial vs precocial) and by egg size in birds [6], [19] as well as by environmental factors such as temperature and oxygen availability [18], [20]. Neurohormonal systems can facultatively modify heart rates [21]; but despite this environmentally-induced flexibility, consistent interspecific differences in heart rates suggest species-specific adaptations [6], [19]. Therefore, comparative studies on heart rates among a phylogenetically diverse sample of species may clarify the ecological underpinnings of adaptation in the metabolic systems of amniote embryos. In the current study, we analyzed relationships between heart rate and other variables (egg size, adult body size, temperature, incubation period) in 54 species of reptiles (including squamates, turtles and crocodilians) to identify intrinsic and extrinsic influences on embryonic heart rates. Our data set allows us to calculate the total number of heart beats during the incubation period, and hence allows us to explore questions about total heart beats during incubation as well as instantaneous heart rates.

## Results

### Thermal dependence of embryonic heart rate

Embryonic heart rate increased as test temperature increased. Q_10_ values of heart rate were greater at low temperatures than at high temperatures (Table 1). Phylogenetically based analyses showed that the temperature effect on Q_10_ values of heart rate was significant in tree 3 (*r* ^2^  =  0.06, *F*_1,77_  =  6.23, *P*  =  0.015) and 4 (*r* ^2^  =  0.06, *F*_1,77_  =  6.00, *P*  =  0.017), close to significant in tree 1 (*r* ^2^  =  0.03, *F*_1,77_  =  3.41, *P*  =  0.069), but non-significant in tree 2 (*r* ^2^  =  0.02, *F*_1,77_  =  2.59, *P*  =  0.112).

**Table 1:** Q_10_ values of embryonic heart rate in different lineages of reptiles, a measure of the rate at which heart rate increased with a 10°C increase in incubation temperature.

	n	20–25°C	25–30°C	30–33.5°C
Lizards	9	2.4±0.2	2.1±0.1	1.9±0.1
Snakes	12	2.3±0.1	2.3±0.1	2.0±0.1
Turtles	5	2.4±0.2	2.2±0.2	1.9±0.2
Crocodiles	1	2.5	2.2	2.0

Q_10_ values of heart rate in embryos were higher at low temperatures than at high temperatures.

### The relationships between embryonic heart rates and adult body mass, egg mass, clutch size and incubation period

**Figure 1:**
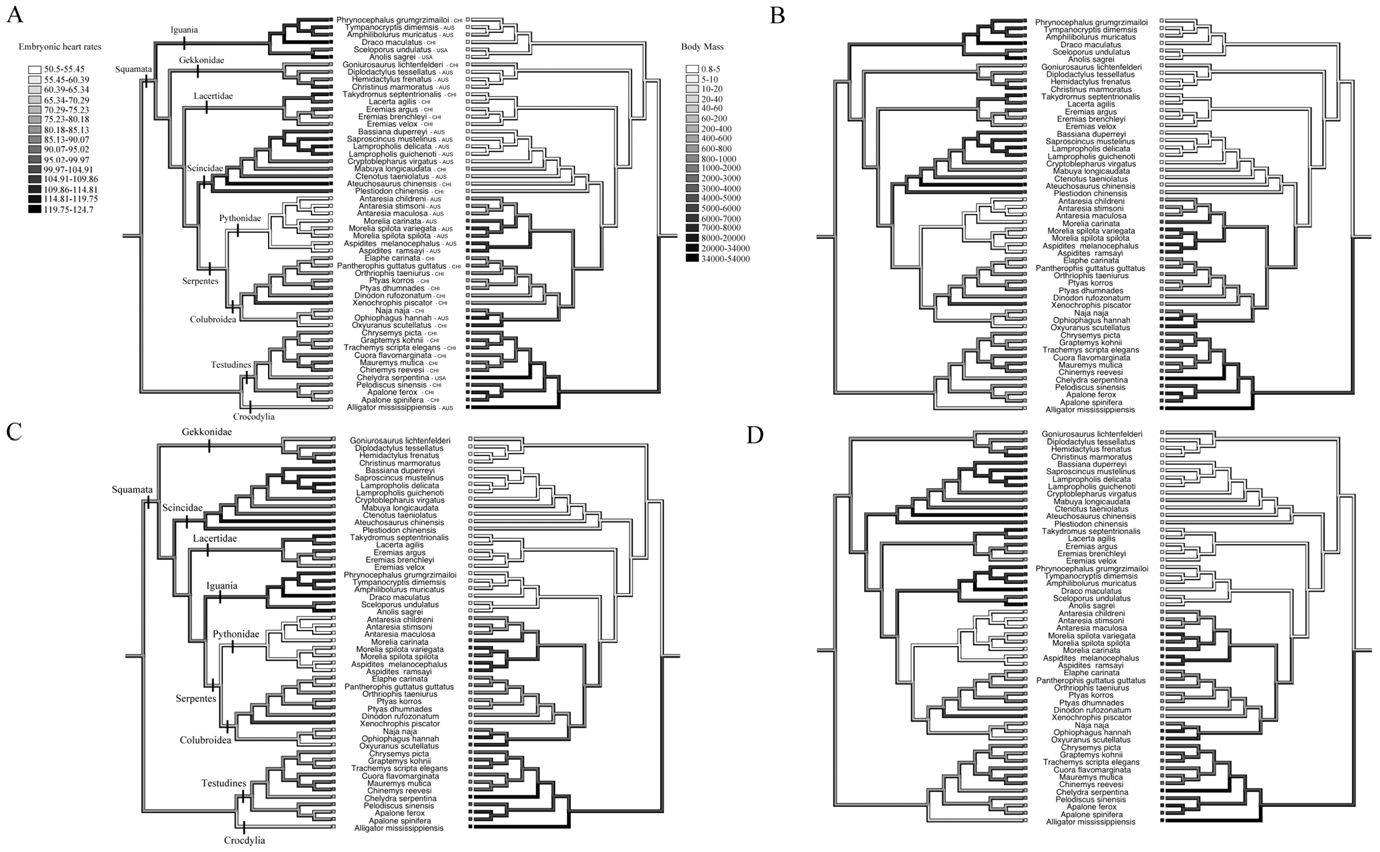
Mirror trees of the evolutionary history reconstructions of embryonic heart rates (beats per minute; left side) and adult body mass (grams; right), according to four different phylogenetic hypotheses (A–D). Some major clades are identified in A (equal positioning in B) and C (equal positioning in D). The three-letter codes after the scientific name of each species indicate the country where heart rate was measured: AUS-Australia, CHI-China, USA-The United States of America.

**Figure 2:**
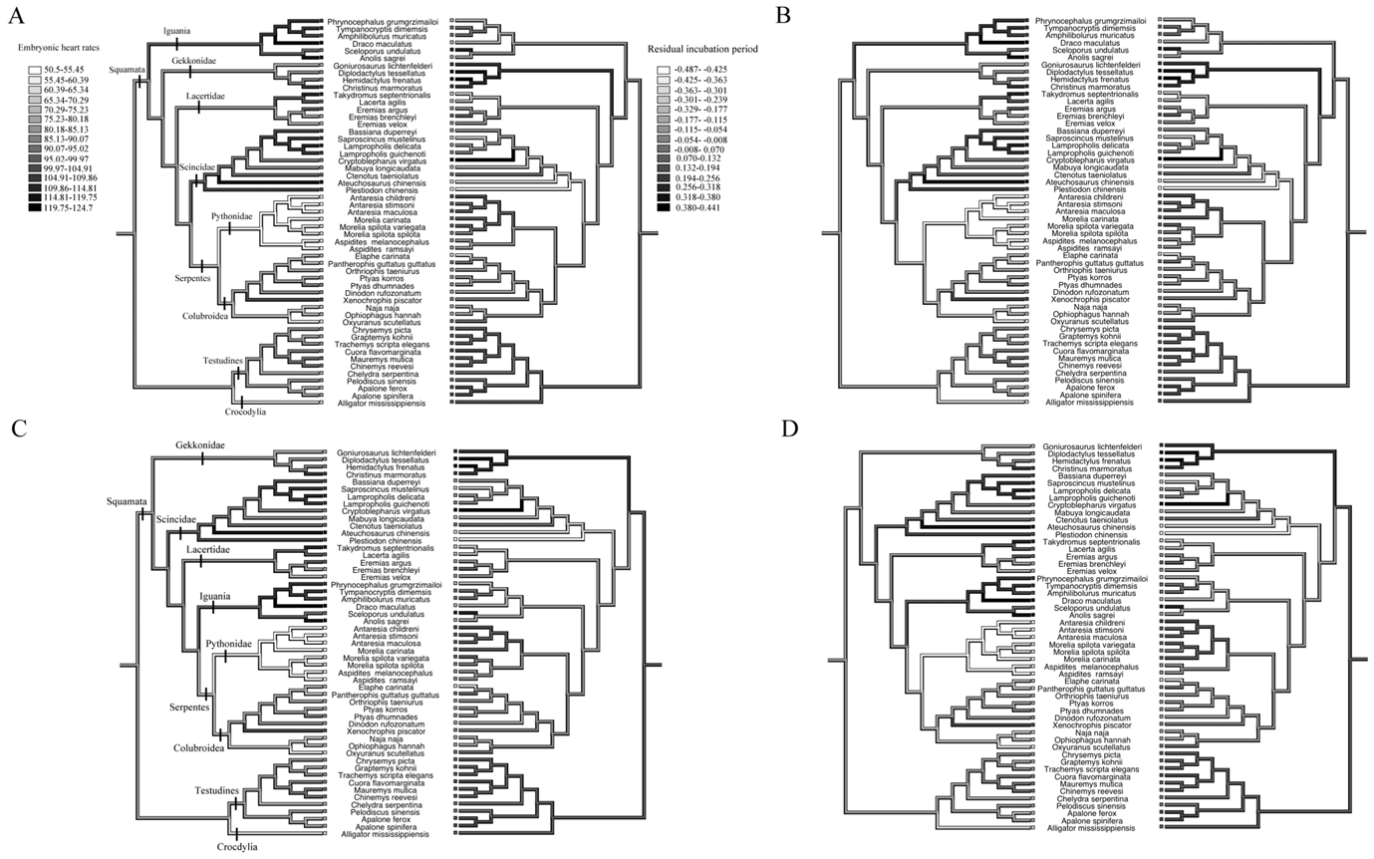
Mirror trees of the evolutionary history reconstructions of embryonic heart rates (beats per minute; left side) and the residual period of incubation (right), according to four different phylogenetic hypotheses (A–D). Some major clades are identified in A (equal positioning in B) and C (equal positioning in D).

Overall, after considering phylogenetic effects in all four trees, embryonic heart rate was negatively related to adult body mass, egg size, incubation period, and clutch size (Table 2). Because incubation period was related to egg mass (*r^2^* > 0.15, *P* < 0.002 for all trees), and clutch size was related to adult body mass (*r^2^* > 0.17, *P* < 0.001 for all trees) we used the residuals of these variables to further analyze the relationship with heart rates. A multiple regression approach showed that embryonic heart rate was negatively correlated with residual incubation period (*P* < 0.00001, for all trees), and adult body mass (*P* < 0.002, for three of the trees), but not residual clutch size (*P* > 0.08, for all trees; Table 3). Our reconstruction of evolutionary changes in these variables shows strong negative correlations between heart rates, adult body mass and residual incubation period among the lizards, snakes (especially within pythons) and crocodilians (Figs. 1, 2).

**Table 2:** Relationships between the standardized phylogenetic contrasts of embryonic heart rate, adult body mass, egg mass, and incubation period in reptiles.

Tree	Adult body mass	Egg mass	Incubation period	Clutch size
1	*r^2^* = 0.15, *F*_1,51_ = 1.29, *P* = 0.001	*r^2^* = 0.19, *F*_1,51_ = 12.06, *P* = 0.0005	*r^2^* = 0.53, *F*_1,51_ = 56.76, *P* < 0.00001	*r^2^* = 0.09, *F*_1,51_ = 5.35, *P* = 0.012
2	*r^2^* = 0.18, *F*_1,51_ = 11.08, *P* = 0.0008	*r^2^* = 0.22, *F*_1,51_ = 14.11, *P* = 0.0002	*r^2^* = 0.54, *F*_1,51_ = 58.91, *P* < 0.00001	*r^2^* = 0.11, *F*_1,51_ = 6.23, *P* = 0.022
3	*r^2^* = 0.14, *F*_1,51_ = 8.38, *P* = 0.003	*R^2^* = 0.17, *F*_1,51_ = 10.56, *P* = 0.001	*r^2^* = 0.52, *F*_1,51_ = 54.55, *P* < 0.00001	*r^2^* = 0.12, *F*_1,51_ = 6.82, *P* = 0.006
4	*r^2^* = 0.16, *F*_1,51_ = 9.91, *P* = 0.001	*r^2^* = 0.19, *F*_1,51_ = 12.31, *P* = 0.0005	*r^2^* = 0.54, *F*_1,51_ = 57.35, *P* < 0.00001	*r^2^* = 0.10, *F*_1,51_ = 5.82, *P* = 0.010

All variables were log-transformed prior analyses. Overall, evolutionary shifts in embryonic heart rate are negatively related to concurrent shifts in adult body mass, egg mass, and incubation period.

**Table 3:** Statistical results of multiple regressions through the origin for the relationships of the standardized phylogenetic independent contrasts of embryonic heart rates, residual incubation time, residual clutch size and adult body mass, according to four alternative phylogenetic hypotheses.

Tree	Residual incubation	Residual clutch size	Adult body mass	Overall multiple regression
1	*t* = −5.12, *P* < 0.00001	*t* = 0.22, *P* = 0.083	*t* = −3.30, *P* = 0.002	*r^2^* = 0.43, *F*_3,49_ = 14.20, *P* < 0.00001
2	*t* = −4.96, *P* < 0.00001	*t* = 0.12, *P* = 0.903	*t* = −3.50, *P* = 0.001	*r^2^* = 0.44, *F*_3,49_ = 14.45, *P* < 0.00001
3	*t* = 5.08, *P* < 0.00001	*t* = −0.68, *P* = 0.497	*t* = 1.27, *P* = 0.209	*r^2^* = 0.33, *F*_3,49_ = 9.68, *P* < 0.0001
4	*t* = −5.18, *P* < 0.00001	*t* = −0.18, *P* = 0.856	*t* = −3.30, *P* = 0.002	*r^2^* = 0.44, *F*_3,49_ = 14.56, *P* < 0.00001

See text for the details of these hypotheses, and of our methods of analysis.

**Figure 3:**
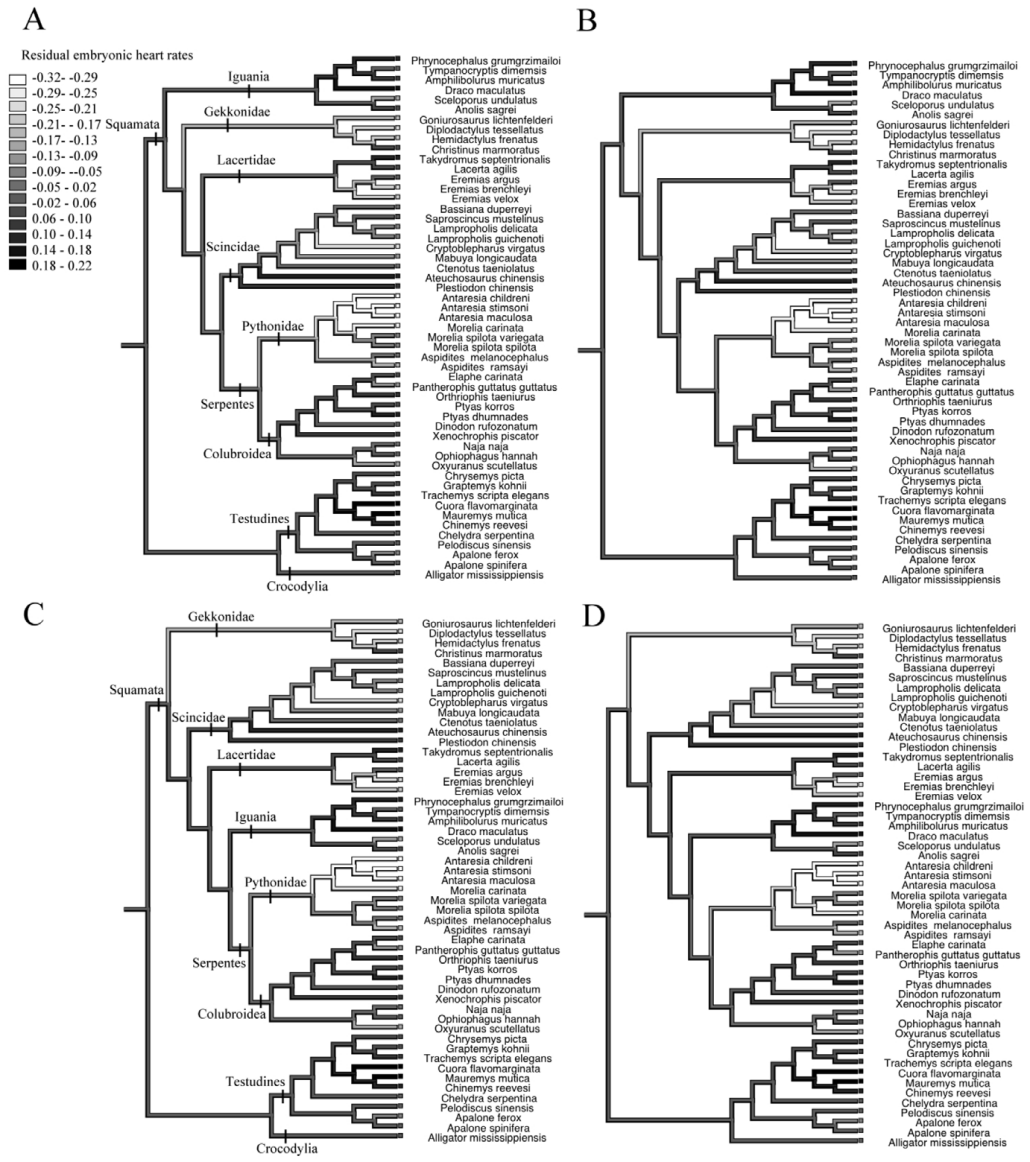
Evolutionary history reconstructions of the residual embryonic heart rates, according to four different phylogenetic hypotheses (A–D). Some major clades are identified in A (equal positioning in B) and C (equal positioning in D).

**Figure 4:**
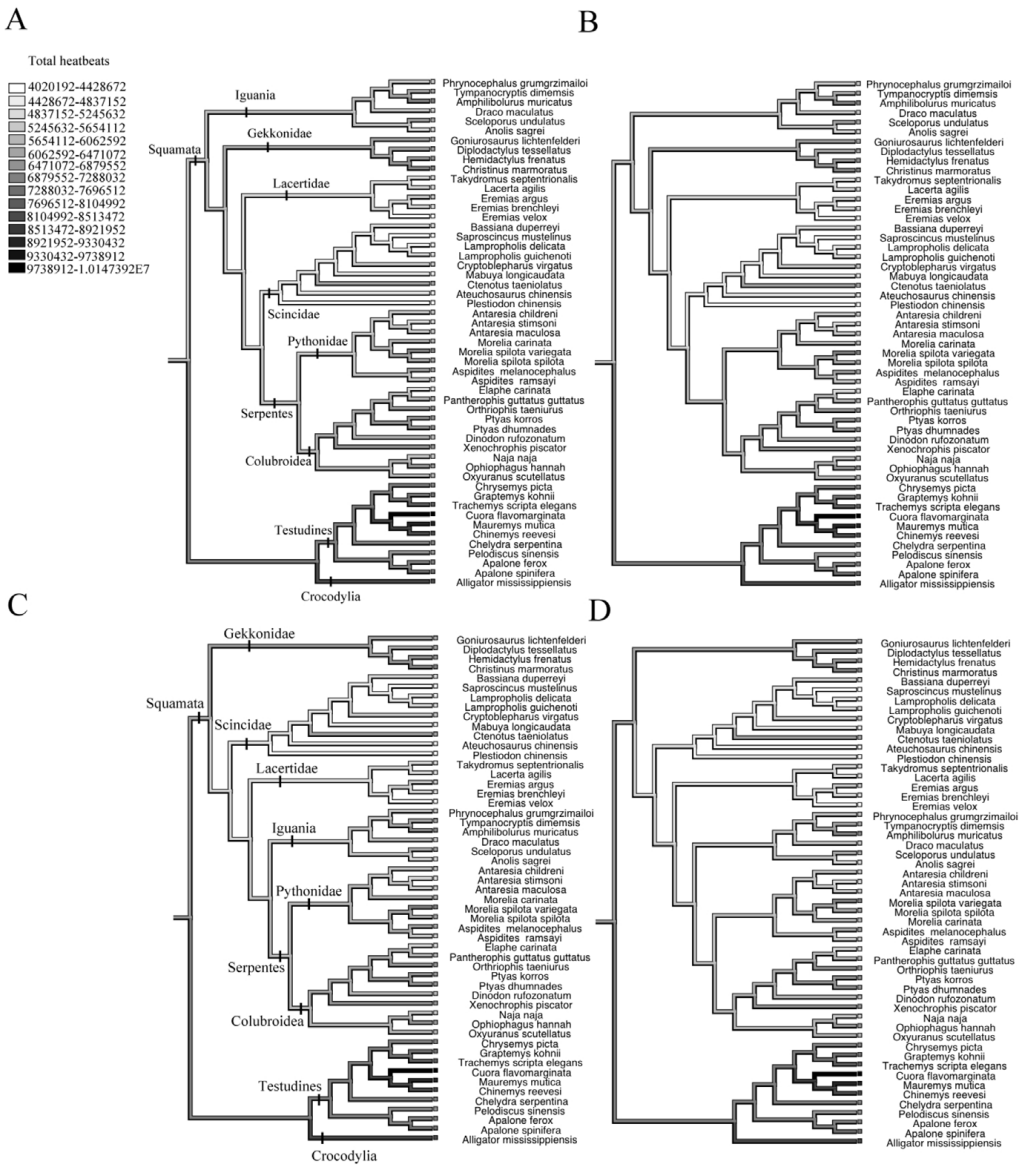
Evolutionary reconstructions of the total number of heart beats during incubation, according to four different phylogenetic hypotheses (A–D). Some major clades are identified in A (equal positioning in B) and C (equal positioning in D).

Mean heart rates also differed among different lineages of reptiles (Figs. 1, 2). However, after the effect of egg size on heart rates was removed the ancestral character reconstruction indicated that most of the variation was among families, especially within the Squamata (Fig. 3). The total number of heart beats required to complete embryonic development at 28°C was positively correlated with egg mass in tree 1 (*r^2^*  =  0.06, *F*_1,51_  =  4.05, *P*  =  0.050), marginally correlated in tree 2 (*r^2^*  =  0.47, *F*_1,51_  =  3.58, *P*  =  0.064), and 4 (*r^2^*  =  0.04, *F*_1,51_  =  3.32, *P*  =  0.074), but not correlated in tree 3 (*r^2^*  =  0.004, *F*_1,51_  =  1.22, *P*  =  0.275). The total number of heart beats differed among lineages: in general, the embryos of crocodiles and turtles required more heart beats prior to hatching than did squamate embryos (Fig. 4). However, there was considerable variation among squamate families (Fig. 4). When the effect of egg mass was removed from the data on total heart rates, the phylogenetic effect at family or subfamily levels was stronger. Species within the Iguania and Gekkonidae required a similar number of heart rates to complete embryonic development as did turtles and crocodilians, whereas other squamates required fewer total heart beats.

## Discussion

The heart rate of a reptile embryo is affected by extrinsic factors such as temperature, as well as by intrinsic lineage-specific and species-specific factors. Below, we consider these in turn.

Embryonic heart rate is highly dependent on environmental temperature because of fundamental biochemical processes (effects of temperature on rates of chemical reactions), as is also the case for other indicators of metabolic rate such as oxygen consumption (both at embryonic and post-embryonic stages [22]). Other extrinsic factors (e.g. moisture, hypoxia) also affect embryonic heart rates, but their effects are minor relative to thermal influences [14], [20], [23]. For example, heart rate in lizards increased twofold when temperature increased 10°C (this study), but did not change when the substrate moisture varied from 0 Kpa to −500 Kpa, and increased by only 20% when the eggs were exposed to hypoxia [11], [20]. Therefore, temperature is likely the most important proximate influence on embryonic heart rate. Even at identical temperatures (e.g. 25°C), however, embryos exhibited a wide range of heart rates – from <40 bpm (pythons) to >100 bpm (anoline lizards). The phylogenetically independent contrast analysis identifies at least three potential correlates of variation in heart rates: adult body mass, egg mass and incubation period.

Allometric relationships between physiological traits (e.g. metabolic rate and heart rate) and body size are widespread in adult animals; typically, mass-specific reaction rates decrease with increasing body size [1], [4], [24]. The dependence of a physiological variable Y on body mass M is often characterized by an equation of the form Y = Y_0_M^b^, where b is the scaling exponent. In birds and mammals, metabolic rates scale as M^3/4^, whereas heart rates scale as M^1/4^ [1], [4], [24]. The current study shows that such allometric relationships also exist in animal embryos: the heart rates (***f_H_***) of embryonic reptiles were significantly related to egg mass(*m_E_*), with larger eggs having embryos with lower heart rates (*f_H_*  =  93.5*m_E_* ^−0.097^; *r^2^*  =  0.61). A similar relationship between heart rate and egg mass is seen in bird embryos also [6], [19]. The regression equations for heart rate *versus* egg mass in these two amniote groups exhibit different constants (93.5 in reptiles vs 371.1 in birds), because of the difference in incubation temperatures of reptiles (mean of 28°C, 50–125 bpm) and birds (mean of 38°C,170–400 bpm). Nonetheless, the scale exponent is similar in reptiles (−0.097) and birds (−0.112) [6]. This allometric similarity suggests that embryonic heart rate is related to egg size in consistent ways in different lineages.

In addition to this general trend, embryonic heart rate shows among-lineage variation. In reptiles, the greatest among-lineage differences in heart rate were among families, especially within the Squamata (Fig. 3). The underlying causes for lineage-specific divergences in embryonic heart rates may include at least three potential factors. First, lineages differ in the embryonic stage at oviposition; some taxa lay their eggs relatively early in embryogenesis, whereas others retain the eggs for longer before laying them [25]. Eggs with less developed embryos at oviposition might benefit from higher heart rates (and therefore, more rapid development) to reduce the potential disadvantage of prolonged incubation. This hypothesis is consistent with evidence from both reptiles and birds. In reptiles, heart rates of turtles and crocodiles (which have relatively undeveloped developed embryos at oviposition [25]) were higher than those of snakes and lizards (which have more developed embryos [25]); and within the Squamata, heart rates of geckos (with lay eggs with less developed embryos) were higher than those of other squamates which lay eggs with more developed embryos (Fig. 3). In bird embryos, similarly, heart rates of precocial taxa are greater than those of altricial taxa [6], [19]. Second, the among-lineage divergence in embryonic heart rates may be associated with phylogenetic divergence in egg size relative to adult body size. Relative egg size may be affected by clutch size (a larger clutch may lead to smaller egg size as a result of the trade-off between egg size and clutch size [26]), and/or by physical constraint (for example, egg size in turtles may be constrained by the width of the pelvic aperture [27]). Third, lineage-specific divergence in embryonic heart rates may be caused by natural selection on the timing of hatching. The fitness of hatchlings may depend upon the time in the season at which the young animal emerges from its egg, with early-emerging offspring having more time to grow before winter, and facing less intraspecific competition [28], [29]. Early hatching is especially important for cold-climate populations experiencing a short activity season, and such populations do indeed enhance developmental rate by increasing embryonic heart rates [7]. A similar phenomenon among species might contribute to the among-lineage differences in heart rate.

The total number of embryonic heart beats during incubation (i.e., from oviposition until hatching) provides a simple index of cardiac function for interspecific comparisons, although this index could be affected by variations of heart rate during incubation (especially during initial phases of embryonic development) [14]. The total number of embryonic heart beats in reptiles varied two fold, from 4.0×10^6^ to 8.5×10^6^, whereas egg mass varied 990-fold from 0.13 g to 128.9 g in our sample. Compared to this dramatic variation in egg mass, the total number of heart beats was relatively invariant. Analogously, several authors have argued that lifespans of post-embryonic stages are characterised by relatively consistent total number of heart beats as well as energy expenditure. The life-span of mammals and birds is roughly fixed when measured in terms of the total number of heart beats (1.47×10^9^ for mammals and 2.32×10^9^ for birds) and in energy expenditure (from 1.4×10^5^ kcal g^−1^ to 2.7×10^5^ kcal g^−1^) across a 50000-fold variation in body mass [1], [6], [30], [31]. Despite this general constancy, the total number of heart beats varies significantly among lineages of oviparous amniotes. Birds generally need more number of heart beats to complete embryogenesis than do reptiles [6], [12]. Within reptiles, turtles and crocodiles require more heart beats to attain the hatching stage than do lizards and snakes.

The among-lineage difference in the total number of embryonic heart beats was related to variation in heart rate and incubation period. Although negatively related to egg mass, heart rate was relatively constant after the effect of egg size was statistically removed in reptiles. This constancy suggests that the among-lineage difference in the total number of heart beats mainly reflects variation in incubation periods. What factors affect incubation period, and thus in turn the total number of heart beats? First, variation in incubation period may stem from different developmental stages of embryos at oviposition. Turtles and crocodiles lay eggs with embryos in early stages of embryogenesis, whereas most squamates complete around 25% of the total period of embryonic development prior to oviposition [25], [32], [33]. This among-lineage difference in developmental stage at oviposition result in about 25% greater number of heart beats being required for embryogenesis during incubation in turtles (mean value 7.7×10^6^) and crocodiles (8.3×10^6^) than in lizards (mean value 5.6×10^6^) and snakes (mean value 5.9×10^6^). Second, variation in incubation period may stem from the developmental degree of offspring at hatching [34]. For example, the absolute number of heart beats required to complete embryogenesis is greater for altricial birds than precocial birds [6]. Although reptiles do not exhibit discrete altricial and precocial reproductive modes, hatching at different developmental degrees may well exist within and among species [35].

In conclusion, the heart rate of a reptilian embryo is affected by both intrinsic and extrinsic factors. Intrinsic factors such as phylogenetic lineage and egg size (in turn determined by adult body size) strongly influence rates of cardiac function during embryogenesis, and generate phylogenetic patterns in embryonic heart rates (and thus, temperature-specific developmental rates [18]). Species-specific physiological adaptations of embryos to their local environments undoubtedly further modify lineage-typical heart rates both via direct selection on developmental rate, and indirect selection mediated via traits such as adult body size and egg size (which themselves are tightly linked to embryonic heart rates). To identify the proximate mechanisms underlying life-history evolution, we cannot afford to ignore the physiology of embryonic development. The increasing availability of methods to non-invasively monitor embryonic function has already revealed an unsuspected ability of embryos to utilize both behavioral and physiological strategies to overcome environmental challenges [9], [18]. The ways in which embryos respond and adapt to biotic and abiotic factors provides exciting opportunities for future studies.

## Materials and Methods

### Egg collection and incubation

We obtained eggs from 54 species of reptiles including 24 taxa of lizards, 18 taxa of snakes, 11 taxa of turtles, and one species of alligator. Most squamate eggs were laboratory-laid by females collected from field, but eggs of pythons, turtles and alligators were mainly collected from commercial hatcheries. We obtained the eggs, and carried out the experiments, in Australia, China and USA. We recorded maximum body mass of females and mean clutch size in most squamates for which females were collected from the field and produced eggs in our laboratory; data on these topics were also extracted from published literature for pythons, turtles and alligators [36], [37], [38]. The number of eggs per species ranged from 2 to 25 (mean  =  13). All eggs were weighed (±0.001 g), and then incubated in moist vermiculite (−200 KPa). Lizard eggs were individually incubated in 64 ml glass jars, whereas clutches of snake, turtle and crocodile eggs were incubated in plastic boxes (135×85×30 mm). The containers were placed into incubators set at constant temperatures suitable for each species (mostly, 28°C).

### Heart rate detection

We measured heart rates of embryos approximately 50 % (range 37 to 62 %) through the total incubation period in all species. As noted above, heart rate is relatively constant throughout embryonic development in reptiles [11], [13], [14], so minor variation in the timing of measurements should have minimal effect on our results. Heart rates (beats per minute, bpm) were measured with an infrared heart rate monitor that detects minute movements of the eggshell (Buddy system, by Avian Biotech; see [12] for details). All eggs were placed in 28 °C incubators for a 2-hour acclimation period prior to measurement, and were then placed individually on the monitor (that was inside the incubator) to record heart rate. To determine the thermal sensitivity of heart rate, eggs of 27 species from different lineages were also measured at 20, 25, 30, and 33.5°C. The total number of heart beats (THB) of an embryo throughout its embryonic development was calculated at any given incubation temperature using the formula THB  =  temperature-specific heart rate × total minutes of developmental time ( =  from oviposition through to hatching) of eggs incubated at that temperature.

All experimental procedures were approved by the Animal Care and Ethics Committee at the University of Sydney (L04/7-2007/3/4665) and Iowa State University (3-09-6695-J), and were conducted in accordance with the NIH *Guide for the Principles of Animal Care*. The experiments on Chinese species were conducted under the ethics license from the University of Sydney, which was accepted by the Animal Care and Ethics Committee at Hangzhou Normal University.

### Phylogenetically–based analyses

We analyzed the data using Felsenstein's [39] independent contrasts analyses, to explore whether or not phylogenetic changes in one variable were consistently associated with changes in another. We compiled previously published phylogenetic hypotheses for groups that included our study species: lizards belonging to the groups Gekkonidae [40], [41], Iguania and Agamidae [42], [43], [44], Lacertidae [45], [46], [47], Scincidae [48], [49], [50], [51]; snakes of the Pythonidae [52], [53], Elapidae [54], [55], Colubridae and Natricidae [56], [57]; and turtles of the Emydidae [58], [59], [60] and Trionychidae [61]. We used the familial-level phylogenetic hypotheses for snakes as proposed by Vidal et al. [57], for turtles by Krenz et al. [62], and the Reptilia by Iwabe et al. [63] and Zardoya and Meyer [64]. Because there are two main conflicting hypotheses for the Squamata [65], [66] and two for the Pythonidae [52], [53], we ran all tests using the four possible combinations of trees. For simplicity we will refer to these hypotheses as ‘tree 1’ (combining the trees by Lee [65] and Kluge [52]), ‘tree 2’ (Lee [65] and Rawlings et al. [53]), ‘tree 3’ (Vidal and Hedges [66] and Rawlings et al.[53]), and ‘tree 4’ (Vidal and Hedges [66] and Kluge [52]).

We reconstructed the ancestral states for the traits we measured (using residuals when traits were correlated – see Results) by linear parsimony in the program Mesquite 2.74 for Macintosh [67], for each tree. The module PDAP:PDTREE 1.15 [68] for Mesquite was used to calculate phylogenetically independent contrasts (PIC; Felsenstein [39] for embryonic heart rates, maximum adult body mass, incubation period, egg mass, clutch size, and total heart rates throughout the incubation period. By combining trees from different authors (who also used different methods for building the trees) we lost information on branch lengths. Thus we used arbitrary lengths calculated in Mesquite, and because different traits may evolve in different rates, branches length differed among traits and trees. In tree 1 and tree 2, for all traits length branches were set using Pagel's method [69]. Then, for the traits maximum body mass, embryonic heart rate, egg mass, clutch size and incubation, the branch to node 2 was multiplied by 0.1(tree 1), and branches to nodes 12, 20, 43, and 103 were multiplied by 3 (tree 1) or 4 (tree 2). For traits Q_10_ (25–30°C) and Q_10_ (30–35°C), the branches to the nodes 2, 3, and 9 were multiplied by −10 in both trees. In tree 3, all branches for maximum body mass, embryonic heart rate, egg mass, clutch size and incubation were set by the method of Grafen [70]. In tree 4, the branches for those traits were transformed by Pagel's methods, and then branches to nodes 8, 21, 35, 64, 92, and 103 were multiplied by 4. In both trees, for Q_10_(20–25°C) all branches were set by Pagel's method, and for Q_10_ (25–30°C) and Q_10_ (30–33.5°C) all branches were set to 1, and branch to node 13 was set to −2.5. Branches lengths for total heart beats were set to 1 in all trees. We used PDAP diagnostic charts to verify that these branch lengths adequately fit the tip data, as required by the PIC analysis [71].

We investigated correlations between the continuous variables by using their standardized PICs in linear regressions through the origin (i.e. intercept equals zero; Felsenstein [39]). Simple regressions were run in PDAP, while multiple regressions were run using the package ‘ape’ 2.3–1 [72] in R 2.12.1 (http://www.R-project.org). Because some of the x-variables were correlated with each other (see results), we first regressed their standardized PICs through the origin, calculated the residuals, and then regressed (through the origin) the residuals against the PICs of the y-variable [71], using the package ‘ape’ in R.
